# Efficacy and safety of thoracic radiotherapy in extensive-stage small-cell lung cancer patients receiving first-line immunotherapy plus chemotherapy: a propensity score matched multicentre retrospective analysis

**DOI:** 10.1186/s13014-024-02420-x

**Published:** 2024-02-27

**Authors:** Yueyuan Yao, Butuo Li, Ruiting Song, Linlin Yang, Bing Zou, Linlin Wang

**Affiliations:** 1 Shandong First Medical University, Shandong Academy of Medical Sciences, Jinan, Shandong 271016 China; 2 Department of Radiation Oncology, Shandong Cancer Hospital and Institute, Shandong First Medical University, Shandong Academy of Medical Sciences, Jiyan Road 440, Jinan, 250117 China

**Keywords:** Extensive-stage small-cell lung cancer, Immune checkpoint inhibitors, Thoracic radiotherapy, Immunotherapy, Chemotherapy, Real-world data

## Abstract

**Background:**

Platinum-etoposide chemotherapy combined with immune checkpoint inhibitors (ICIs) has been recommended as the first-line standard treatment for extensive-stage small-cell lung cancer (ES-SCLC). However, the effect of thoracic radiotherapy (TRT) on these patients is still unknown. This study aimed to evaluate the efficacy and safety of TRT for ES-SCLC patients who responded to first-line ICIs and chemotherapy (CHT).

**Methods:**

Patients who received 4 to 6 cycles of ICIs and CHT as first-line therapy at three hospitals between 2018 and 2022 were included in the analysis. All patients were divided into two groups based on whether they received TRT as first-line treatment, and propensity score matching (PSM) was performed to ensure that the characteristics of two groups were well-balanced. The primary endpoints were overall survival (OS) and progression-free survival (PFS), and the secondary endpoint was toxic effects.

**Results:**

A total of 276 patients were included, and the median follow-up time was 22.3 (range, 4.0-53.73) months. After PSM, 197 patients were further analysed, and 99 of whom received TRT. The baseline characteristics were well-balanced between patients in the TRT and non-TRT groups. There were significant differences in PFS between the TRT and non-TRT groups, with the median PFS of 10.76 and 7.63 months, respectively (P = 0.014). Significantly improved OS was observed in the TRT group (21.67 vs. 16.6 months, P = 0.009). In addition, the use of TRT was an independent prognostic factor for PFS and OS of ES-SCLC patients receiving ICIs plus CHT. In terms of safety, no significant increase of any grades adverse event (AE) (P = 0.874) and G3-4 AE (P = 0.909) was observed for patients receiving TRT. Radiation esophagitis, gastrointestinal and hematologic toxicities were the most common AEs in TRT group, which were tolerable. And high-dose radiotherapy was associated with higher incidence of pneumonitis.

**Conclusion:**

Addition of TRT showed significant survival benefits and well tolerability in ES-SCLC patients receiving platinum-etoposide CHT and ICIs, which could be a feasible first-line treatment strategy for ES-SCLC patients.

**Supplementary Information:**

The online version contains supplementary material available at 10.1186/s13014-024-02420-x.

## Introduction

Small-cell lung cancer (SCLC) accounts for 13–17% of lung cancer and is characterized by rapid proliferation, aggressive growth, and early widespread metastasis [1, 2]. Approximately two-thirds of patients with SCLC are classified as extensive-stage SCLC (ES-SCLC) at initial diagnosis. Four to six cycles of platinum-based chemotherapy (CHT) are the cornerstone of the treatment for ES-SCLC. However, the survival of ES-SCLC patients is poor, with the median progression-free survival (PFS) of less than 5 months and overall survival (OS) of less than 9 months [3]. Several studies investigating other approaches for ES-SCLC treatment, such as radiotherapy, targeted drugs, and immunotherapy, have been performed [4–14].

The notable improvement in survival from atezolizumab was showed based on the IMpower133 trial [4], with the median PFS of 5.2 months and OS of 12.3 months. Survival benefits from immune checkpoint inhibitors (ICIs) were also observed in the CASPIAN, CAPSTONE-1 and ASTRUM-005 trials [5–7]. Thus, the combination of ICIs and CHT has been recommended as the standard first-line treatment strategy for ES-SCLC.

Radiotherapy (RT) plays an important role in all stages of disease presentation, especially in intrathoracic tumour control after first-line treatment. Previous studies have shown that the combination of thoracic radiotherapy (TRT) with CHT could decrease local recurrence-free survival rates and prolong OS in ES-SCLC patients compared to CHT alone [15–17]. The CREST trial showed 10% improvement in 2-year survival rate in patients submitted to TRT after responding to first-line CHT [18]. Moreover, several retrospective studies have indicated that TRT combined with CHT is related to long-term survival [14, 17, 19–21]. Thus, TRT is recommended for patients with ES-SCLC in NCCN and ASTRO guidelines [22, 23].

In the era of immunotherapy, RT has been proven to remodulate the immune microenvironment and to have synergistic effects with ICIs [24, 25]. Adding radiotherapy to pembrolizumab immunotherapy has been found to significantly increase responses and outcomes in patients with metastatic non-small cell lung cancer (NSCLC) [26]. A real-world study has demonstrated favorable survival and good tolerability of the combination of PD-1/PD‐L1 inhibitors plus palliative radiotherapy in metastatic NSCLC patients [27]. However, the effect of applying TRT to ES-SCLC patients who receiving ICIs is unclear. Whether the combined TRT with ICIs and CHT can further improve the treatment efficacy without significantly increasing toxicity is worth further investigation.

This multicentre retrospective analysis is carried out with the intention of evaluating the efficacy and safety of TRT for ES-SCLC patients who responded to first-line ICIs and CHT.

## Materials and methods

### Patients

This is a retrospective cohort study. Patients who were histologically confirmed ES-SCLC at three hospitals from July 2018 to October 2022 were included in this study. All participants meeting the following criteria were eligible for this study: (1) pathologically confirmed SCLC; (2) radiological confirmed extensive-stage SCLC according to the AJCC TNM staging system(stage IV [T any, N any, M1a or M1b]) [28], or Veterans Administration Lung Study Group(VALG) staging system (T3-4 due to multiple lung nodules that are too extensive or tumour/nodal volume that is too large to be encompassed in a tolerable radiation plan) [29]; (3) receiving at least 4 cycles of immunotherapy plus CHT as the first-line treatment; (4) without progression after 4 treatment cycles; and (5) have accurate clinical follow-up data. The exclusion criteria as follows: (1) patients with limited SCLC disease; (2) patients who progressed after first-line therapy; (3) previously received TRT. All enrolled patients were divided into two groups according to whether they received TRT during the first-line treatment.

### Treatment strategy

All patients received CHT combined with ICIs. The CHT regimens consisted primarily of platinum and etoposide, while the ICIs included PD-1/L1 inhibitors. Patients who received TRT after 4 cycles of CHT and ICI treatment were assigned to the TRT group. The radiotherapy treatment plan was either 3-dimensional conformal radiotherapy (3D-CRT) or intensity-modulated radiotherapy (IMRT). The gross tumour volume (GTV) included the residual primary tumour after CHT-ICIs treatment and the positive lymph nodes before CHT-ICIs treatment. The clinical target volume (CTV) was defined as the gross tumour volume (GTV) with a margin of 5 mm and positive lymph node drainage areas. The planning target volume (PTV) was expanded from the CTV with a margin of 5 to 8 mm. Given the different dose fractionations regimens of radiotherapy, we used the biological effective dose (BED) formula: BED = nd×[1 + d/(α/β)] [30–32], where n is the fraction of radiotherapy, d represents the dose per fraction, and α/β is the ratio of radiosensitivity coefficients.

### Data records and assessment

Clinical characteristics for analysis included patients age, gender, smoking history, Eastern Cooperative Oncology Group performance status (ECOG PS), histological type, tumour stage, baseline metastasis sites, immunotherapy and chemotherapy regimens, thoracic radiotherapy data, and survival data. Clinical assessments were performed by Response Evaluation Criteria in Solid Tumors (RECIST) version 1.1 [33]. Response assessment was performed every other cycle during immunotherapy, and every 6–8 weeks post-treatment until disease progression. PFS was defined as the time from initial treatment to disease progression or death or last follow-up, and OS defined as the time from initial treatment to death of any cause or last follow-up. The best overall response was defined as the best response during the first-line treatment setting. AEs were assessed and graded by the senior doctors according to the Common Terminology Criteria for Adverse Events version 5.0 [34]. Receiver operating characteristic (ROC) curve was performed to evaluate the predictive effect of radiotherapy dose (BED) and volumes on the AEs. And Square test or Fisher’s exact test was performed to explore the associations between the radiation dose and volume and AEs.

### Statistical analysis

Propensity score matching (PSM) (1:1) was performed to ensure that the patients characteristics between the TRT and non-TRT groups were well-balanced. Square test or Fisher’s exact test was performed to compare the baseline characteristics between different groups. Kaplan-Meier method and log-rank tests were used for survival analysis. The hazard ratios (HR) and 95% confidence interval (95% CI) for OS and PFS were estimated by stratified Cox regression model. All statistical analyses were two-tailed tests and P value < 0.05 was considered statistically significant. The statistical analysis was carried out with IBM SPSS 26.0.

## Results

### Patient characteristics

From July 2018 to October 2022, a total of 276 patients from three hospitals were enrolled in analysis. The characteristics of the study population are summarized in Table 1. The median age was 62 (range, 41-80) years old. Most patients were men (82%), and 159 (58%) patients had a history of smoking. A total of 187 patients (68%) received PD-L1 inhibitors (atezolizumab and durvalumab), while the other 89 patients (32%) received PD-1 inhibitor s (serplulimab, tislelizumab, etc.). 117 (42%) of them received TRT in the first-line treatment, whereas 159 (58%) did not. After PSM, 197 patients in total were further analyzed, and 99 of whom received TRT. Before receiving TRT, 67.7% of patients had a partial response (PR) or complete response (CR) in the TRT group. The ORR was 70.4% in the non-TRT group after chemotherapy and ICIs. In addition, 32.3% and 29.6% patients showed stable disease (SD) in the TRT and non-TRT groups, respectively. Baseline characteristics were well balanced between patients receiving TRT or not.

**Table 1:** Baseline characteristics of ES-SCLC patients before and after PSM

**Characteristic**	Before matching			Characteristic	After matching		
	TRT Group (n = 117)	non-TRT Group (n = 159)	P value		TRT Group (n = 99)	non-TRT Group (n = 98)	P value
Age, years			0.363	Age, years			0.939
< 60	55(47.0%)	66(41.5%)		< 60	46(46.5%)	45(45.9%)	
≥60	62(53.0%)	93(58.5%)		≥60	53(53.5%)	53(54.1%)	
Gender			0.135	Gender			0.492
Male	92(78.6%)	136(85.5%)		Male	78(78.8%)	81(82.7%)	
Female	25(21.4%)	23(14.5%)		Female	21(21.2%)	17(17.3%)	
Smoking history			0.486	Smoking history			0.821
Yes	65(55.6%)	95(59.7%)		Yes	56(56.6%)	57(58.2%)	
No	52(44.4%)	64(40.3%)		No	43(43.4%)	41(41.8%)	
ECOG PS			0.08	ECOG PS			0.418
0–1	92(78.6%)	110(69.2%)		0–1	74(74.7%)	78(79.6%)	
2	25(21.4%)	49(30.8%)		2	25(25.3%)	20(20.4%)	
T stage			0.909	T stage			0.832
T1-T2	61(52.1%)	84(52.8%)		T1-T2	48(48.5%)	49(50.0%)	
T3-T4	56(47.9%)	75(47.2%)		T3-T4	51(51.5%)	49(50.0%)	
N stage			0.387	N stage			0.434
N0-N2	61(52.1%)	73(45.9%)		N0-N2	53(53.5%)	47(48.0%)	
N3	56(47.9%)	86(54.1%)		N3	46(46.5%)	51(52.0%)	
M stage			**0.002**	M stage			0.665
M1a	24 (20.5%)	21 (13.2%)		M1a	19 (19.2%)	17 (17.4%)	
M1b	72 (61.5%)	80 (50.3%)		M1b	60 (60.6%)	56 (57.1%)	
M1c	21 (18.0%)	58 (36.5%)		M1c	20 (20.2%)	25 (25.5%)	
Type of ICIs			0.652	Type of ICIs			0.793
PD-1	36(30.8%)	53(33.3%)		PD-1	31(31.3%)	29(29.6%)	
PD-L1	81(69.2%)	106(66.7%)		PD-L1	68(68.7%)	69(70.4%)	
NO. of metastatic sites			**0.004**	NO. of metastatic sites			0.837
≤ 2	106(90.6%)	123(77.4%)		≤ 2	88(88.9%)	88(89.8%)	
> 2	11(9.4%)	36(22.6%)		> 2	11(11.1%)	10(10.2%)	
Brain metastases			0.295	Brain metastases			0.725
Yes	40(34.2%)	45(28.3%)		Yes	33(33.3%)	35(35.7%)	
No	77(65.8%)	114(71.7%)		No	66(66.7%)	63(64.3%)	
Liver metastases			**< 0.001**	Liver metastases			0.899
Yes	24(20.5%)	66 (41.5%)		Yes	24(24.2%)	23(23.5%)	
No	93(79.5%)	93(58.5%)		No	75(75.8%)	75(76.5%)	
Bone metastases			**0.039**	Bone metastases			0.596
Yes	27(23.1%)	55(34.6%)		Yes	24(24.2%)	27(27.6%)	
No	90(76.9%)	104(65.4%)		No	75(75.8%)	71(72.4%)	
Response evaluation*			0.794	Response evaluation*			0.758
CR/PR	81(69.2%)	107(67.3%)		CR/PR	67(67.7%)	69(70.4%)	
SD	36(30.8%)	52(32.7%)		SD	32(32.3%)	29(29.6%)	

PSM, propensity score matching; ECOG PS, Eastern Cooperative Oncology Group Performance Status; ICIs, immune checkpoint inhibitors; NO., number; TRT, thoracic radiotherapy

*Response evaluation before TRT or immunotherapy maintenance

The total dose of radiation ranges from 30 to 66 Gy, with a median dose of 50 Gy and median BED of 60 Gy. The median PTV volume was 197.3 cm^3^. Out of 99 patients who received TRT, 58 received conventional fractionated TRT (44-66 Gy/1.8-2.1 Gy/22-30f, BED = 36.0-79.2 Gy), 29 received hypofractionated TRT (36-60 Gy/2.5-3 Gy/12-24f, BED = 39.0-62.5 Gy), and the rest 12 patients received hyperfractionated TRT (30-60 Gy/1.5 Gy/20-40f, BED = 34.5-69.0 Gy) (Supplementary Table 1). Besides, there were 8.1% patients receiving PCI.

### Survival outcomes and treatment response

The median follow-up time was 22.3 (range, 4.0-53.7) months at the time of data cut-off. A total of 132 patients (67%) experienced disease progression, and 119 patients (60.4%) died from any cause. The median PFS and OS were 9.17 and 17.70 months, respectively, in the whole population.

**Fig. 1:**
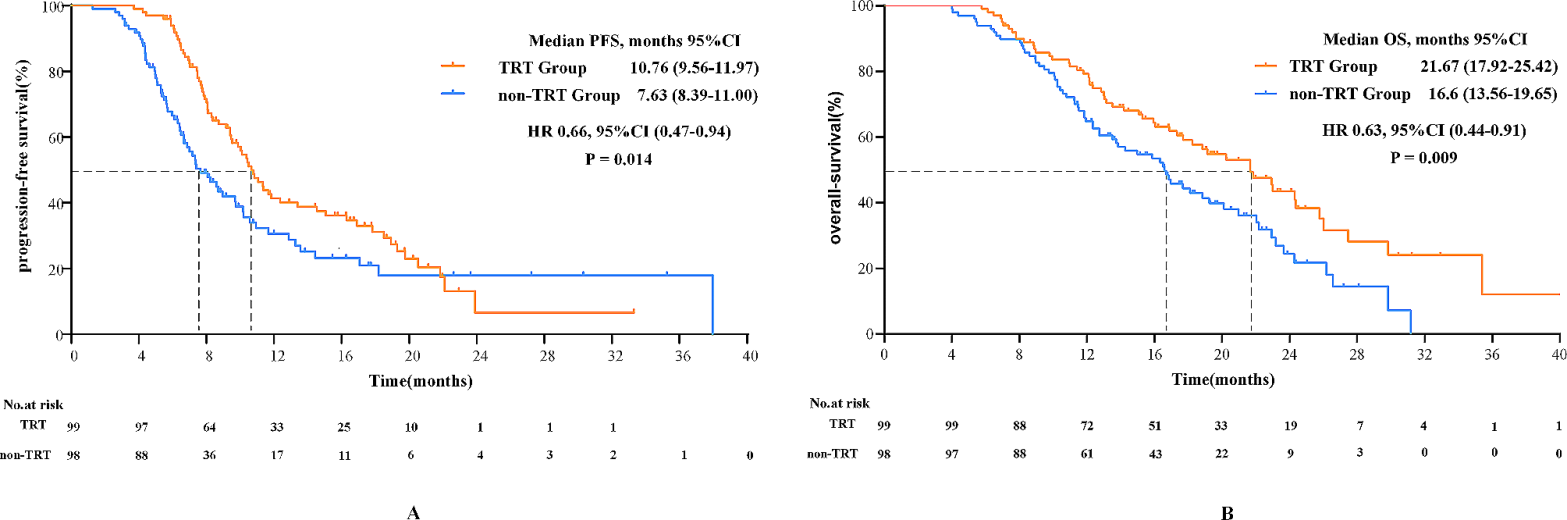
Kaplan–Meier curves of (A) PFS and (B) OS between ES-SCLC patients in TRT or non-TRT groups. PFS, progression-free survival; OS, overall survival; TRT, thoracic radiotherapy; CI, confidence interval; HR, hazard ratio

Survival analysis indicated that patients who received TRT in the first-line setting had better PFS than patients who did not receive TRT (median PFS: 10.76 months vs. 7.63 months; P = 0.014) after matching (Fig. 1A). The estimated 1-year PFS rate was 41.9% versus 30.6% in the TRT group and non-TRT group, respectively (Table 2).

**Table 2:** Survival estimate of patients in the TRT and non-TRT group

Survival estimated	TRT Group (n = 99)	non-TRT Group (n = 98)	P value
**1-year PFS, %**	41.9	30.6	0.030
**1-year OS, %**	78.2	64.8	0.033
**2-year OS, %**	41.5	24.5	0.019

PFS, progression-free survival; OS, overall survival; TRT, thoracic radiotherapy

Prolonged survival was observed in TRT group with the median OS of 21.67 months compared to 16.60 months in non-TRT group (HR, 0.63; 95% CI, 0.44-0.91; P = 0.009) (Fig. 1B). And the 1-year survival rate was 78.2% versus 64.8%, and 2-year survival rate was 41.5% versus 24.5% in the TRT group and non-TRT group, respectively.

**Fig. 2:**
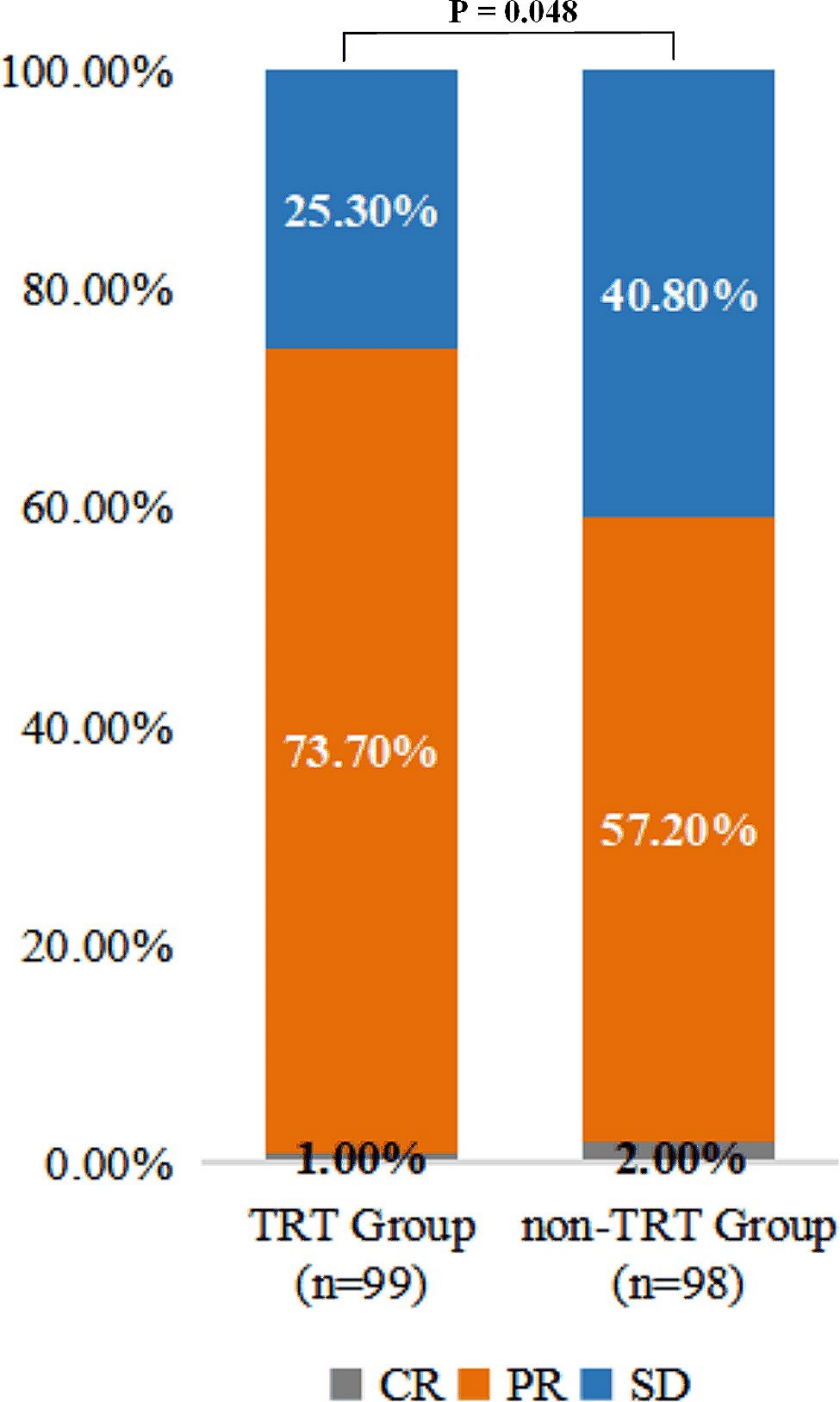
The comparison of best overall response between patients in TRT and non-TRT group. CR, complete response; PR, partial response; SD, stable disease; PD, progressive disease; ORR, objective response rate; TRT, thoracic radiotherapy

In total, 73.7% patients had a partial response (PR) in TRT group, 57.1% in non-TRT group. Additionally, 25.3% vs. 40% patients showed stable disease (SD) in TRT group and non-TRT group, respectively. The objective response rate (ORR) was 74.7% in TRT group, which was considerably higher than that in non-TRT group (59.2%, P = 0.048) (Fig. 2).

### Subgroup analysis of survival outcomes

**Fig. 3:**
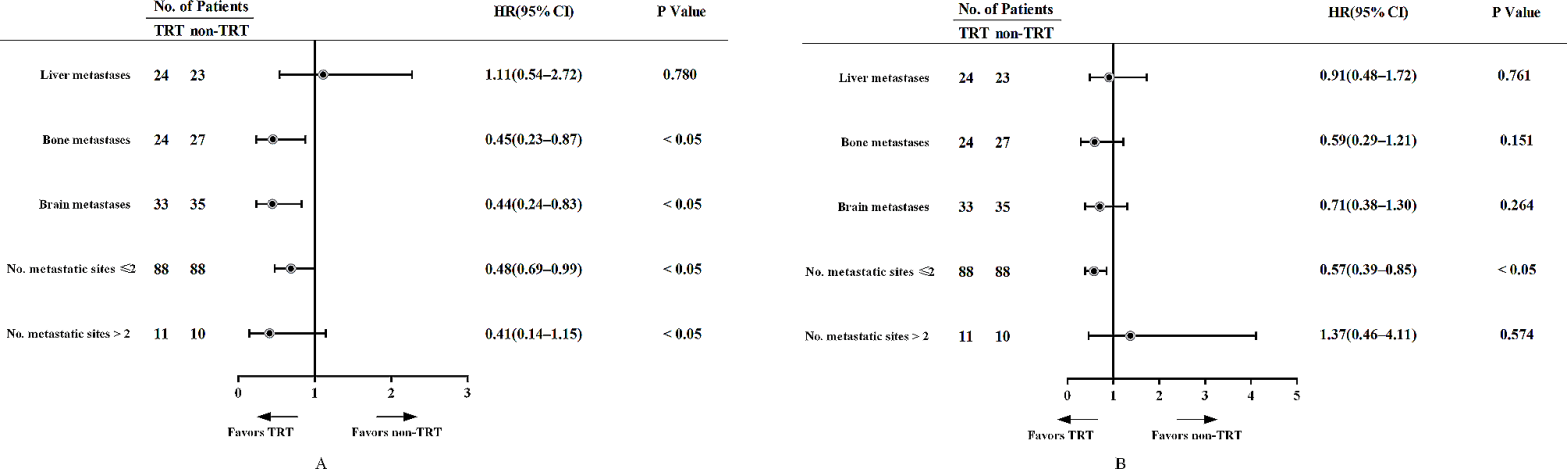
Subgroup analysis of survival outcomes in TRT and non-TRT group. TRT, thoracic radiotherapy; No., number; HR, hazard ratio; CI, confidence interval

Subgroup analysis was performed based on the number of metastatic sites and site of metastases, and the results are shown in Fig. 3. The addition of TRT was beneficial for both PFS and OS in ES-SCLC patients with ≤ 2 metastatic sites but not in patients with > 2 metastatic sites. Besides, patients with liver metastasis did not achieve clinical benefit from TRT. The superiority of TRT in PFS but not OS was shown in patients with bone or brain metastases.

Patients with bone metastases, brain metastases, or distant metastases who received TRT could reduce the risk of disease progression (Fig. 3A). Patients with liver metastases, bone metastases, brain metastases, or metastatic sites ≤ 2 who received TRT had a decreased risk of death (Fig. 3B). However, only those metastatic sites ≤ 2 patients received TRT with significant differences.

### Univariate and multivariate cox analyses of PFS and OS

Univariate Cox analysis revealed that male, T3-T4 stage, N3 stage, M1c stage, PD-L1 inhibitors, and not receiving TRT were significantly related to shorter PFS. Multivariate analysis demonstrated that male, T3-T4 stage, M1c stage, PD-L1 inhibitors, and not receiving TRT were independent factors for worse PFS (Table 3). In terms of OS, univariate analysis revealed that age ≥ 60 years, M1b stage, M1c stage, liver metastases, metastases sites > 2, and not receiving TRT were associated with poor OS. Multivariate analysis showed that age ≥ 60, M1b stage, M1c stage, liver metastases, and not receiving TRT were independent poor prognostic factors of poor OS (Table 4).

**Table 3:** Univariate and multivariate cox analyses of PFS for ES-SCLC patients receiving ICIs

Variables	Univariate analysis		Multivariate analysis	
	HR (95% CI)	P value	HR (95% CI)	P value
Gender				
Male				
Female	0.608 (0.390–0.946)	0.027*	0.586 (0.374–0.917)	0.019*
Age				
≥ 60				
< 60	0.999 (0.707–1.410)	0.994		
Smoking History				
Yes				
No	0.785 (0.552–1.116)	0.177		
ECOG PS				
2				
≥ 2	1.266 (0.845–1.898)	0.253		
T stage				
≤ 2				
> 2	1.603 (1.132–2.272)	0.008*	1.778 (1.240–2.550)	0.002*
N stage				
≤ 2				
> 2	1.596 (1.121–2.270)	0.009*	1.436 (0.998–2.066)	0.051
M stage				
M1a				
M1b	1.096 (0.681–1.764)	0.705		
M1c	1.866 (1.706–3.293)	0.026*	1.956 (1.117–3.424)	0.019*
Type of ICIs				
PD-1				
PD-L1	1.718 (1.156–2.554)	0.007*	1.684 (1.120–2.533)	0.012*
Receiving TRT				
No				
Yes	0.651 (0.460–0.920)	0.015*	0.599(0.420–0.854)	0.005*
NO. of metastatic sites				
< 3				
≥ 3	1.605 (0.917–2.808)	0.098		
Liver metastases				
No				
Yes	1.192 (0.798–1.780)	0.391		
Bone metastases				
No				
Yes	1.409 (0.964–2.060)	0.077		
Brain metastases				
No				
Yes	1.039 (0.719–1.502)	0.838		

PFS, progression-free survival; CI, confidence interval; ECOG PS, Eastern Cooperative Oncology Group Performance Status; ICIs, immune checkpoint inhibitors; TRT, thoracic radiotherapy; NO., number; HR, hazard ratio; CI, confidence interval; *P < 0.05

**Table 4:** Univariate and multivariate cox analyses of OS for ES-SCLC patients receiving ICIs

Variables	Univariate analysis		Multivariate analysis	
	HR (95% CI)	P value	HR (95% CI)	P value
Gender				
Male				
Female	0.802 (0.507–1.266)	0.343		
Age				
≥ 60				
< 60	0.661 (0.457–0.956)	0.028*	0.593 (0.408–0.862)	0.006*
Smoking History				
Yes				
No	0.835 (0.580–1.202)	0.332		
ECOG PS				
< 2				
≥ 2	1.208 (0.791–1.845)	0.382		
T stage				
≤ 2				
> 2	1.335 (0.929–1.920)	0.118		
N stage				
≤ 2				
> 2	1.249 (0.869–1.794)	0.230		
M stage				
M1a				
M1b	1.994 (1.153–3.449)	0.014	2.051 (1.163–3.620)	0.013*
M1c	3.516 (1.878–6.583)	< 0.001*	2.818 (1.437–5.527)	0.003*
Type of ICIs				
PD-1				
PD-L1	0.996 (0.676–1.466)	0.983		
Receiving TRT				
No				
Yes	0.618 (0.428–0.892)	0.010*	0.670 (0.464–0.966)	0.032*
NO. of metastatic sites				
≤ 2				
> 2	1.883 (1.070–3.314)	0.028*	1.836 (0.939–3.592)	0.076
Liver metastases				
No				
Yes	2.007 (1.364–2.953)	**< 0.001**	1.722 (1.160–2.558)	0.007*
Bone metastases				
No				
Yes	1.094 (0.726–1.648)	0.669		
Brain metastases				
No				
Yes	1.247 (0.849–1.829)	0.260		

OS, overall survival; CI, confidence interval; ECOG PS, Eastern Cooperative Oncology Group Performance Status; ICIs, immune checkpoint inhibitors; TRT, thoracic radiotherapy; NO., number; HR, hazard ratio; CI, confidence interval; *P < 0.05

### Safety

The toxicity profiles of the combination of TRT and CHT plus immunotherapy are shown in Tables 5 and 6. Most of adverse events (AEs) were tolerable and self-limiting, which were easily handled and managed. Leucopenia/white-cell count decreased was the most common G1-2 AEs (44.4%). Radiation esophagitis was the second common G1-2 AEs (38.4%) in patients receiving TRT, and the third G1-2 AEs was nausea (27.3%). Neutropenia (8.1%) and pneumonitis (6.1%) were the most common and second common G3-4 AEs, respectively. G3-4 radiation esophagitis developed 4% of patients. Only one patient developed grade 4 pneumonitis leading to radiotherapy withdrawal. No grade 5 adverse events occurred.

**Table 5:** Comparison of G1-2 adverse events between TRT and non-TRT group

Adverse Events	TRT Group (n = 99)	non-TRT Group (n = 98)	P value
Any Grades	82 (82.8%)	82 (83.7%)	0.874
G1-2 Totally	78 (78.8%)	78 (79.6%)	0.889
Leucopenia /White-cell count decreased	44 (44.4%)	34 (34.7%)	0.190
Neutropenia /Neutrophil count decreased	23 (23.2%)	21 (21.4%)	0.864
Thrombocytopenia /Platelet count decreased	7 (7.1%)	2 (2.0%)	0.170
Anaemia	5 (5.1%)	13 (13.3%)	0.051
Nausea	27 (27.3%)	25 (25.5%)	0.872
Decreased appetite	17 (17.2%)	28 (28.6%)	0.063
Constipation	9 (9.1%)	14 (14.3%)	0.276
Diarrhea	6 (6.1%)	7 (7.1%)	0.783
Radiation esophagitis	38 (38.4%)	/	/
Pneumonitis	16 (16.2%)	9 (9.2%)	0.199
Myocarditis	0 (0%)	0 (0%)	/
Hypothyroid	1 (1.0%)	1 (1.0%)	1.000
Atrial fibrillation	0 (0%)	0 (0%)	/

G1-2: grade 1 to 2

**Table 6:** Comparison of G3-4 adverse events between TRT and non-TRT group

Adverse Events	TRT Group (n = 99)	non-TRT Group (n = 98)	P value
Any Grades	81 (81.8%)	82 (83.7%)	0.874
G3-4 Totally	28 (28.3%)	27 (27.6%)	0.909
Leucopenia /White-cell count decreased	4 (4.0%)	2 (2.0%)	0.683
Neutropenia /Neutrophil count decreased	8 (8.1%)	12(12.2%)	0.356
Thrombocytopenia /Platelet count decreased	1 (1.0%)	2 (2.0%)	0.621
Anaemia	1 (1.0%)	2 (2.0%)	0.621
Nausea	4 (4.0%)	7 (7.1%)	0.373
Decreased appetite	1 (1.0%)	3 (3.1%)	0.369
Constipation	0 (0%)	0 (0%)	/
Diarrhea	0 (0%)	2 (2.0%)	0.246
Radiation esophagitis	4 (4.0%)	/	/
Pneumonitis	6 (6.1%)	3 (3.1%)	0.498
Myocarditis	3 (2.0%)	0 (0%)	0.497
Hypothyroid	1 (1.0%)	0 (0%)	1.000
Atrial fibrillation	0 (0%)	1 (1.0%)	0.497

G3-4: grade 3 to 4

Compared with the non-TRT group, there was no significantly increased any grades AEs (P = 0. 874) and G3-4 AEs (P = 0.909) for patients receiving TRT. The incidence of G1-2 pneumonitis was proportionally higher in the TRT group (16.2%) compared to non-TRT group (9.2%), but the difference was not statistically significant (P = 0.199). And G3-4 pneumonitis was 6.1% versus 3.1% in two groups, respectively (P = 0.498).

Receiver operating characteristic (ROC) curves were plotted to determine the relationship between BED, PTV volume and incidence of AE in the TRT group, with a cut-off of 62.45 Gy and 209 cm^3^. Patients were classified into low-dose (BED ≤ 62.45 Gy) and high-dose (BED > 62.45 Gy), based on a predetermined cut-off value by ROC. Significantly higher pneumonitis was observed for patients high-dose group compared to low-dose group (34.2% vs. 14.8%, P = 0.028). Radiation esophagitis, and hematologic toxicities were more common in the high-dose group but no statistical significance was observed. (Supplementary Table 2). There were no significant differences on high grade toxicities between patients in high-dose and low-dose group (Supplementary Table 2). Besides, higher PTV volume was associated with increased incidence of radiation esophagitis and hematologic toxicities, but not pneumonitis and gastrointestinal toxicities (Supplementary Table 3). And, the volume of PTV was not associated with the incidence of grade 3-4 AEs.

## Discussion

Nowadays, ICIs combined with CHT have been recommended as the standard first-line treatment option for ES-SCLC patients [35]. Although ES-SCLC patients always experience a high objective response to first-line systemic therapy, most patients progress or die rapidly from recurrence, metastasis, and drug-resistance [5–7, 36]. Thus, we intended to seek treatment modalities to improve efficacy and prolong survival of ES-SCLC patients. The results of the present study indicated prolonged survival and acceptable AEs with the addition of TRT in the first-line treatment of patients with ES-SCLC receiving ICIs plus CHT.

CHT could stimulate tumour antigen expression, priming the tumour for response to ICIs. In addition, pre-clinical evidence has demonstrated that synergistic immune stimulation against cancer cells from incorporating RT and immunotherapy [37]. RT can increase antigen presentation, promote T cell infiltration, and favorably modulate the tumour microenvironment [24, 38, 39], which would amplify immune response and improve efficacy when combined with ICIs [40]. Given the evidence of preclinical data and the benefit of RT in local control of lung cancer, the combination treatment of RT and ICIs is recommended in clinical practice [23]. Previous reports have suggested the clinical benefit from combination treatment in local advanced and metastatic NSCLC patients [26, 41–44]. However, the efficacy and safety of this treatment strategy are largely unknown in ES-SCLC. Diamond et al. performed a single-arm retrospective study enrolling 20 patients and found favorable safety profile and improved OS (median OS) with the use of TRT and ICIs plus CHT in the management of ES-SCLC [45]. Another single-arm retrospective analysis included 36 patients also suggested that combined with TRT in the first-line treatment was safe and had ample survival benefits [46].

To our knowledge, two ongoing randomized trials (RAPTOR trial and TREASURE study) evaluating the safety and efficacy of the addition of TRT for ES-SCLC patients receiving first-line ICIs plus chemotherapy, and no results have been reported yet. Our investigation is the first multicentre, head-to-head study to compare the efficacy and safety of ES-SCLC patients treated with TRT and ICIs-CHT in the first-line setting. The results of this study showed the 5-and 11-month improvement of PFS and OS from the addition of TRT respectively, which indicated efficacy from the addition of TRT. And the subgroup analysis indicated the obvious benefit from the TRT in oligometastatic ES-SCLC patients. However, the addition of TRT was not recommended for patients with liver metastasis.

In terms of safety, no significantly increase in AEs was observed for patients receiving TRT in this study. A single-arm phase II trial of 21 patients cross China showed that the combination of low-dose radiotherapy was tolerable in patients with ES-SCLC (NCT04622228) [47]. Additionally, a phase I trial (NCT02402920) conducted at MD Anderson, which included 38 patients, demonstrated that TRT-pembrolizumab was well tolerated, with low grade 3 and greater toxic events [48].

Esophagitis, gastrointestinal and hematologic toxicities were the common AEs associated with the addition of TRT for ES-SCLC patients receiving ICI and CHT. In the CREST trial, the frequency of G3-4 esophagitis was 1.6% [18], which is lower than our study (4%) and a real-world retrospective study (8.3%) [14]. The possible reason for this result might to be related to the differences in the dose of TRT, with a segment of patients in our research received a dose of 50-60 Gy compared to 30 Gy in the CREST trial. Another real-world retrospective investigation included ES-SCLC patients receiving CHT and TRT with a dose of 40-60 Gy, and the results indicated that 8.3% of the patients developed G3-4 esophagitis [14].

Pneumonitis as an adverse event with high mortality requires great attention. The PACIFIC study indicated that no significantly increased risk of pneumonitis observed with the combination of TRT and ICIs plus CHT (33.9% vs. 24.8%) for NSCLC patients [41]. Notably, the real-world studies have indicated that the incidence of pneumonitis is 7 -19% for NSCLC patients receiving ICIs and CHT [49–55], which is higher than that reported in clinical trials. The higher incidence of pneumonitis in real-world studies was also observed for NSCLC patients receiving TRT and ICIs plus CHT [50, 56]. The heterogeneity of general conditions and ethnic groups in real-world settings versus clinical trials may contribute to the different incidences of pneumonitis [57–59]. In our study, the incidence of pneumonitis in ICIs plus CHT group is 12.2%, which was higher than the results of the clinical trial (4-8.2%) [5–7]. Moreover, there was no statistically significant increase in the incidence of pneumonitis with the addition of TRT (22.2% vs. 12.2%, respectively, P = 0.089) in the present study. It should be noted that the radiation dose was significantly associated with the incidence of pneumonitis. In summary, these data suggest a favorable safety profile of adding TRT to ICIs plus CHT in first-line treatment of ES-SCLC patients. And high-dose TRT was associated with the higher incidence of pneumonitis.

In this study, there were several limitations. First, as a retrospective cohort analysis, there was potential selection bias. Though we performed a multicentre analysis to minimize the impact of possible confounding factors, the study findings need to be interpreted cautiously. Second, due to the retrospective nature of the study, some heterogeneities emerged in the study population, such as different cycles of CHT plus ICIs, TRT techniques and doses et. As a result of these flaws, further large phase III prospective studies are needed to validate the results of our findings.

## Conclusion

In conclusion, the addition of TRT showed significant survival benefits and well tolerability in ES-SCLC patients receiving platinum-etoposide chemotherapy and ICIs. The results suggest that TRT plus CHT and ICIs could be a feasible first-line treatment strategy for ES-SCLC patients but should be investigated in further studies, such as the ongoing RAPTOR trial and TREASURE study.

### Electronic supplementary material

Below is the link to the electronic supplementary material.

Supplementary Material 1

## Data Availability

The data that support the findings of this study are available from the corresponding author, [LL.W], upon reasonable request.

## Abbreviations

ICIs: immune checkpoint inhibitors
ES-SCLC: extensive-stage small-cell lung cancer
TRT: thoracic radiotherapy
CHT: chemotherapy
OS: overall survival
PFS: progression-free survival
AE: adverse event
3D-CRT: 3-dimensional conformal radiotherapy
IMRT: intensity-modulated radiotherapy
BED: biological effective dose
PTV: planning target volume
GTV: gross tumour volume
CTV: clinical target volume
ROC: receiver operating characteristic
