# Supplementary material for: Efficacy and safety of thoracic radiotherapy in extensive-stage small-cell lung cancer patients receiving first-line immunotherapy plus chemotherapy: a propensity score matched multicentre retrospective analysis
Source: Radiat Oncol. 2024 Feb 27;19:25. doi: 10.1186/s13014-024-02420-x (PMC10900720; doi:10.1186/s13014-024-02420-x)
Supplement: Supplementary file 1 — Supplementary Material 1 [file 13014_2024_2420_MOESM1_ESM.docx]

| **Supplementary Table 1** Characteristics of thoracic radiotherapy regimes. | |
| --- | --- |
| **Radiotherapy information** | |
| Radiation dose, median Gy (IQR) | 50 (45-56) |
| Biological effective dose (BED), median Gy (IQR) | 60 (55.2-67.2) |
| Sequence of TRT and CHT, n (%) |  |
| Sequential | 71 (71.7) |
| Concurrent | 28 (28.3) |
| Radiation Techniques, n (%) |  |
| IMRT | 95 (96.0) |
| 3D-CRT | 4 (4.0) |
| Radiation fractionation, n (%) |  |
| Once daily | 87 (87.9) |
| Twice daily | 12 (12.1) |
| Radiation Dose, n (%) |  |
| Conventional fractionation | 58 (58.6) |
| Hyperfractionation | 12 (12.1) |
| Hypofractionation | 29 (29.3) |
| PTV volume (cm^3^), n (%)  ≤ 209 cm^3^  > 209 cm^3^ | 53 (53.5)  46 (46.5) |
| PCI, n (%) | 8 (8.1) |

TRT, thoracic radiotherapy; IMRT, intensity-modulated radiation therapy; PTV, planning target volume;

3D-CRT, 3D conformal radiotherapy; PCI, prophylactic cranial irradiation.

**Supplementary Table 2** Adverse events summarized by the BED of TRT.

| **Adverse Events, n (%)** | **High-dose group (n = 38)  (BED > 62.45 Gy)** | **Low-dose group (n = 61) (BED ≤ 62.45 Gy)** | **P value** |
| --- | --- | --- | --- |
| **All grade AEs** |  |  |  |
| Pneumonitis | 13 (34.2) | 9 (14.8) | **0.028** |
| Radiation esophagitis | 20 (52.6) | 22 (36.1) | 0.143 |
| Hematologic toxicities^a^ | 23 (60.5) | 30 (49.2) | 0.305 |
| Gastrointestinal toxicities^b^ | 14 (36.8) | 24 (39.3) | 0.835 |
| **Grade 3-4 AEs** |  |  |  |
| Pneumonitis | 4 (10.5) | 3 (4.9) | 0.423 |
| Radiation esophagitis | 3 (7.9) | 1 (1.6) | 0.295 |
| Hematologic toxicities^a^ | 10 (26.3) | 10 (16.4) | 0.304 |
| Gastrointestinal toxicities^b^ | 3 (7.9) | 12 (19.7) | 0.152 |

TRT, thoracic radiotherapy; BED, biological effective dose; AE, adverse event.

^a^Hematologic toxicities include leucopenia, neutropenia, thrombocytopenia, and anaemia.

^b^Gastrointestinal toxicities include nausea, decreased appetite, constipation, and diarrhea.

**Supplementary Table 3** Adverse events summarized by PTV volume of TRT

| **Adverse Events, n (%)** | **PTV Volume > 209 cm^3^  (n = 46)** | **PTV Volume ≤ 209 cm^3^ (n = 53)** | **P value** |
| --- | --- | --- | --- |
| **All grade AEs** |  |  |  |
| Pneumonitis | 10 (21.7) | 12 (22.6) | 0.914 |
| Radiation esophagitis | 30 (65.2) | 12 (22.6) | **< 0.001** |
| Hematologic toxicities^a^ | 30 (65.2) | 23 (43.4) | 0.043 |
| Gastrointestinal toxicities^b^ | 18 (39.1) | 20 (37.7) | 0.887 |
| **Grad 3-4 AEs** |  |  |  |
| Pneumonitis | 3 (6.5) | 4 (7.5) | 0.843 |
| Radiation esophagitis | 3 (6.5) | 1 (1.9) | 0.335 |
| Hematologic toxicities^a^ | 8 (17.4) | 12 (22.6) | 0.619 |
| Gastrointestinal toxicities^b^ | 5 (10.9) | 10 (18.9) | 0.400 |

PTV, planning target volume; TRT, thoracic radiotherapy; AE, adverse event.

^a^Hematologic toxicities include leucopenia, neutropenia, thrombocytopenia, and anaemia.

^b^Gastrointestinal toxicities include nausea, decreased appetite, constipation, and diarrhea.
